# The relationship between clinical characteristics, sociodemographic factors and sedentary behavior in adults with chronic conditions: a cross-sectional study from the Lolland-Falster Health Study

**DOI:** 10.1186/s11556-026-00407-z

**Published:** 2026-04-13

**Authors:** Lars Bo Jørgensen, Sofie Rath Mortensen, Lars Hermann Tang, Alexander Harrison, Anders Grøntved, Jan Christian Brønd, Randi Jepsen, Therese Lockenwitz Petersen, Søren T. Skou

**Affiliations:** 1 Faculty of Health, Department of Midwifery, Physiotherapy, Occupational Therapy and Psychomotor Therapy, University College Copenhagen, Copenhagen, Denmark; 2 The Research and Implementation Unit PROgrez, Department of Physiotherapy and Occupational Therapy, Næstved-Slagelse-Ringsted Hospitals, Slagelse, Denmark; 3 Department of Physiotherapy and Occupational Therapy, Zealand University Hospital, Roskilde, Denmark; 4 Research Unit for Musculoskeletal Function and Physiotherapy, Department of Sports Science and Clinical Biomechanics, University of Southern Denmark, Odense, Denmark; 5 The Research Unit for Exercise Epidemiology, Centre of Research in Childhood Health, Department of Sports Science and Clinical Biomechanics, University of Southern Denmark, Odense, Denmark; 6 Department of Regional Health Research, University of Southern Denmark, Odense, Denmark; 7 Department of Health Sciences, University of York, Heslington, York, York, UK; 8 Lolland-Falster Health Study, Zealand University Hospital, Nykøbing Falster, Denmark; 9 Steno Diabetes Center, Copenhagen, Denmark

**Keywords:** Accelerometer, Sedentary time, Sedentary bout length, Multimorbidity, Lolland-Falster Health study

## Abstract

**Background:**

Sedentary behavior (SB) increases the risk of disease and mortality, even in physically active individuals. Adults living with multimorbidity may be particularly vulnerable. Although factors such as age, body mass index (BMI), and social support have been suggested, evidence is largely based on self-reported activity that tends to underestimate SB; therefore, correlates of objectively measured SB in adults with medical conditions remain insufficiently described. We investigated clinical and sociodemographic correlates of accelerometer-measured SB among adults with medical conditions, including multimorbidity.

**Methods:**

We used backwards stepwise regression to investigate associations between clinical characteristics, sociodemographic factors, and SB in Danish adults (≥ 18 years) with ≥ 1 medical condition and valid accelerometer data from the Lolland-Falster Health Study (*n* = 1,728). Multimorbidity was present in 963 participants. SB was measured using accelerometers and expressed as daily sedentary time and weighted median sedentary bout length.

**Results:**

BMI, number of medical conditions, civil and employment status, sex, and mental well-being were associated with sedentary time and/or weighted median sedentary bout length. Obesity class III was associated with 1.43 more sedentary hours/day compared with underweight/normal weight, and having three or ≥ 4 medical conditions was associated with 0.30 and 0.28 more sedentary hours/day, respectively, compared with one medical condition. Living alone was associated with 0.30 more sedentary hours/day and 8.50 seconds longer sedentary bout length compared with being married/living with a partner. Being unemployed/other or retired was associated with 0.64 and 0.39 more sedentary hours/day, respectively, compared with paid employment. Women spent 0.23 fewer sedentary hours/day than men, and reduced mental well-being was associated with 0.23 more sedentary hours/day compared with moderate-to-high mental well-being.

**Conclusion:**

Several clinical and sociodemographic factors were associated with SB in adults with medical conditions. These results can inform the identification of groups who may benefit from strategies to reduce sedentariness, including people living with multimorbidity.

**Supplementary Information:**

The online version contains supplementary material available at 10.1186/s11556-026-00407-z.

## Background

Sedentary behavior (SB) is characterized by periods with low energy expenditure [1], and has emerged as a significant public health concern [2], due to its link to an increased risk of disease and all-cause mortality regardless of the individual’s physical activity (PA) level [3–5].

While evidence from interventions aiming to reduce SB remains limited [6, 7], emerging evidence suggests that even small increases in PA and breaks from prolonged sitting may benefit health outcomes [8, 9]. Thus, health benefits can be obtained by replacing SB with PA, which has become a cornerstone of contemporary health promotion [10].

In adults with medical conditions, reducing SB may be an important target for health promotion. SB has been associated with multimorbidity [11–15], and multimorbidity often requires complex and individualized treatment plans [16]. An improved understanding of factors related to SB therefore may help tailor strategies to reduce sedentariness, including for people living with medical conditions.

Determinants and correlates of SB in adults and older adults have received increased attention over recent years. Systematic reviews suggest that individual-level factors such as higher age, sex, increased body mass index (BMI), and lower socioeconomic status have been reported to be associated with higher SB in adults and older adults [17, 18]. SB is also associated with multimorbidity, and a higher multimorbidity burden has been linked to higher sedentary time [11].

Importantly, self-reported instruments tend to underestimate sedentary time compared with device-based methods [19]. Therefore, studies using objective measurements are warranted to improve estimates of associations between clinical and sociodemographic factors and SB in adults with medical conditions, including those living with multimorbidity, as evidence based on objective measurement methods in these populations remains relatively limited [20–22]. We therefore aimed to investigate correlates of accelerometer-measured SB among adults with medical conditions, including people living with multimorbidity.

## Methods

### Study design

This study was designed as a cross-sectional study and reported in accordance with the Strengthening the reporting of observational studies in epidemiology (STROBE) guidelines [23] (Supplementary file 1).

### Data sources and setting

Data were derived from the Lolland-Falster Health Study (LOFUS), a Danish household-based population study. LOFUS data collection included questionnaires, health examinations, and biological samples and was conducted between February 2016 and February 2020. Questionnaire development and data collection in LOFUS have been described elsewhere [24, 25].

From February 2017 to November 2018, LOFUS participants who were able to walk and belonged to families with at least one adult and one child were invited to take part in an accelerometer sub-study as part of a cross-sectional investigation of PA resemblance in families [26]. From December 2018 to February 2020, recruitment to accelerometer measurement was expanded to include all LOFUS participants with walking ability [27].

### Study population

All adults (≥ 18 years) with valid accelerometer data and at least one self-reported medical condition (≥ 1) were included. A valid accelerometer measurement was defined as ≥ 22 hours of wear time/day, including ≥ 10 hours of awake time/day, with at least three valid weekdays and one valid weekend day; data were weighted 5/7 for weekdays and 2/7 for weekends. The final analytical sample comprised 1,728 participants, of whom 963 met the definition of multimorbidity.

Multimorbidity was defined as ≥ 2 medical conditions affecting ≥ 2 body systems, determined using a classification procedure described previously [28, 29] (Supplementary file 2). The ten body systems were: lung, musculoskeletal, endocrine, mental, cancer, neurological, gastrointestinal, cardiovascular, kidney, and sensory organs.

### Exposures

Clinical characteristics (BMI, number of medical conditions, mental well-being, and chronic pain) and sociodemographic factors (age, sex, civil status, socioeconomic status (SES), and employment status) were included as exposure variables and to describe participant characteristics. These variables were selected a priori based on documented associations with SB in adult populations [17–19, 30, 31] and their availability in the LOFUS dataset.

BMI was calculated using the formula; kg/m^2^. Height was measured using a SECA 216 wall-mounted stadiometer and weight using a Tanita Body Composition Analyzer (BC-420MA III) or an electronic scale (Tanita WB 150 SMA) during the health examination [24]. BMI was categorised as underweight/normal weight (< 25.0), overweight (25.0–29.99), obesity class I (30.0–34.99), obesity class II (35.0–39.99), and obesity class III (≥ 40.0), in accordance with World Health Organization definitions [32].

The number of medical conditions was derived from self-reported questionnaire data and categorised into four groups: (1) 1 medical condition, (2) 2 medical conditions, (3) 3 medical conditions, and (4) ≥ 4 medical conditions. Consistent with our multimorbidity classification, categories 2–4 represent conditions affecting at least two, three, and ≥ 4 different body systems, respectively. A similar categorisation has been used previously [29].

Mental well-being was assessed using the self-reported WHO-5 Well-Being Index [33], consisting of five items rated from 0 (“at no time”) to 5 (“all of the time”), with higher scores indicating better well-being. The raw score was multiplied by 4 to yield a total score ranging from 0 to 100, as recommended. Scores < 50 were classified as reduced mental well-being and scores ≥ 50 as moderate-to-high mental well-being [34].

Chronic pain was assessed using self-reported questionnaire data and defined as pain lasting ≥ 6 months (yes/no).

Age and sex were obtained from the Danish Civil Registration System (CPR). Age was defined at the date of the health examination and categorised according to recommendations by Geifman et al. [35] as 18–44, 45–64, 65–79, and ≥ 80 years. Sex was categorised as male or female.

Civil status was derived from questionnaire data by combining responses to (1) legal marital status and (2) whether the participant lived with a partner (cohabiting). Responses were collapsed into a binary variable: (1) married/living with a partner or (2) living alone.

Socioeconomic status (SES) was operationalised as self-reported educational level and categorised as: <10 years (primary), 10–12 years (upper secondary or vocational), ≥ 13 years (higher education), or other.

Employment status was self-reported at the time of questionnaire completion. Responses were categorised as (1) in paid employment (employed; employee and self-employed; self-employed; apprentice/trainee), (2) unemployed/other (unemployed; student; vocational rehabilitation; sick leave ≥ 3 months; military service; assisting spouse; stay-at-home parent; other), or (3) retired (voluntary early retirement; early retirement due to reduced work capacity; age-based retirement).

### Outcomes

SB was assessed using two accelerometers (Axivity AX3, Newcastle, UK) worn continuously for seven consecutive days (24 hours/day), including during water activities [27], with one device placed on the right thigh and one on the lower back. Data processing has been described previously [26] and the measurement procedure has been validated [36, 37].

Based on recommendations by Byrom et al. [38], the primary outcomes were (1) total sedentary time and (2) weighted median sedentary bout length. Total sedentary time was defined as the mean time (hours/day) spent in a sitting, reclined, or lying position during the day (07:00–22:00) and was derived using both accelerometers. Weighted median sedentary bout length was defined as the bout duration (seconds) at which 50% of total sedentary time is accumulated when sedentary bouts are ordered from shortest to longest.

For descriptive purposes, additional SB and PA metrics were calculated: total active time, number of sedentary bouts, and fragmentation index. Total active time was defined as time spent in PA (min/day) at light, moderate, or vigorous intensity during the day (07:00–22:00) and was derived from the lower-back accelerometer. A sedentary bout was registered when a participant was classified as sitting, reclined, or lying for ≥ 10 seconds; number of sedentary bouts was the total count per day. The fragmentation index was calculated as the number of sedentary bouts divided by total sedentary time (minutes).

### Statistical methods

Before initiating the analyses, a statistical analysis plan (SAP) was developed and made publicly available via Open Science Framework (https://osf.io/zw2t6/?view_only=6690b774bdde49258b4d6559fa2f3918). The SAP specified the included variables and planned analyses.

For the present study, the analytical sample was restricted to participants with ≥ 1 medical condition. Multimorbidity was defined as ≥ 2 medical conditions affecting ≥ 2 body systems. Number of medical conditions was modelled categorically with 1 medical condition as the reference group.

Descriptive statistics for clinical characteristics, sociodemographic factors, and accelerometer-derived SB and PA metrics were summarised for all participants with ≥ 1 medical condition and presented overall and stratified by 1 medical condition versus multimorbidity (≥ 2). Continuous variables are presented as mean (SD) or median (IQR), as appropriate, and categorical variables as n (%).

We inspected the distribution of the outcome variables visually using histograms and Q–Q plots. Based on these inspections, observations with total sedentary time < 4 hours/day and weighted median sedentary bout length < 100 seconds were considered outliers and excluded; seven observations were removed.

Associations between candidate correlates and each outcome were examined using linear regression. A stepwise approach with backward elimination was applied starting from a full model including BMI, number of medical conditions, mental well-being, chronic pain, age, sex, civil status, SES (educational level), and employment status. At each step, variables were retained if *p* < 0.05. Age and sex were forced into all models a priori as key sociodemographic variables: age given its well-established relationship with accumulation of medical conditions/multimorbidity [39], and sex based on evidence suggesting potential associations with SB [17].

Because accelerometer recruitment in LOFUS involved household-based enrolment, analyses accounted for clustering at the household level (household ID). Model assumptions were evaluated using diagnostic plots. As predictors in the final models were entered as categorical variables, linearity of continuous predictors was not applicable to the reported models. Two final models were fitted (one per outcome), and results are presented as regression coefficients (β) with 95% confidence intervals and p-values. All analyses were conducted using Stata/BE 18.0.

## Results

**Fig. 1:**
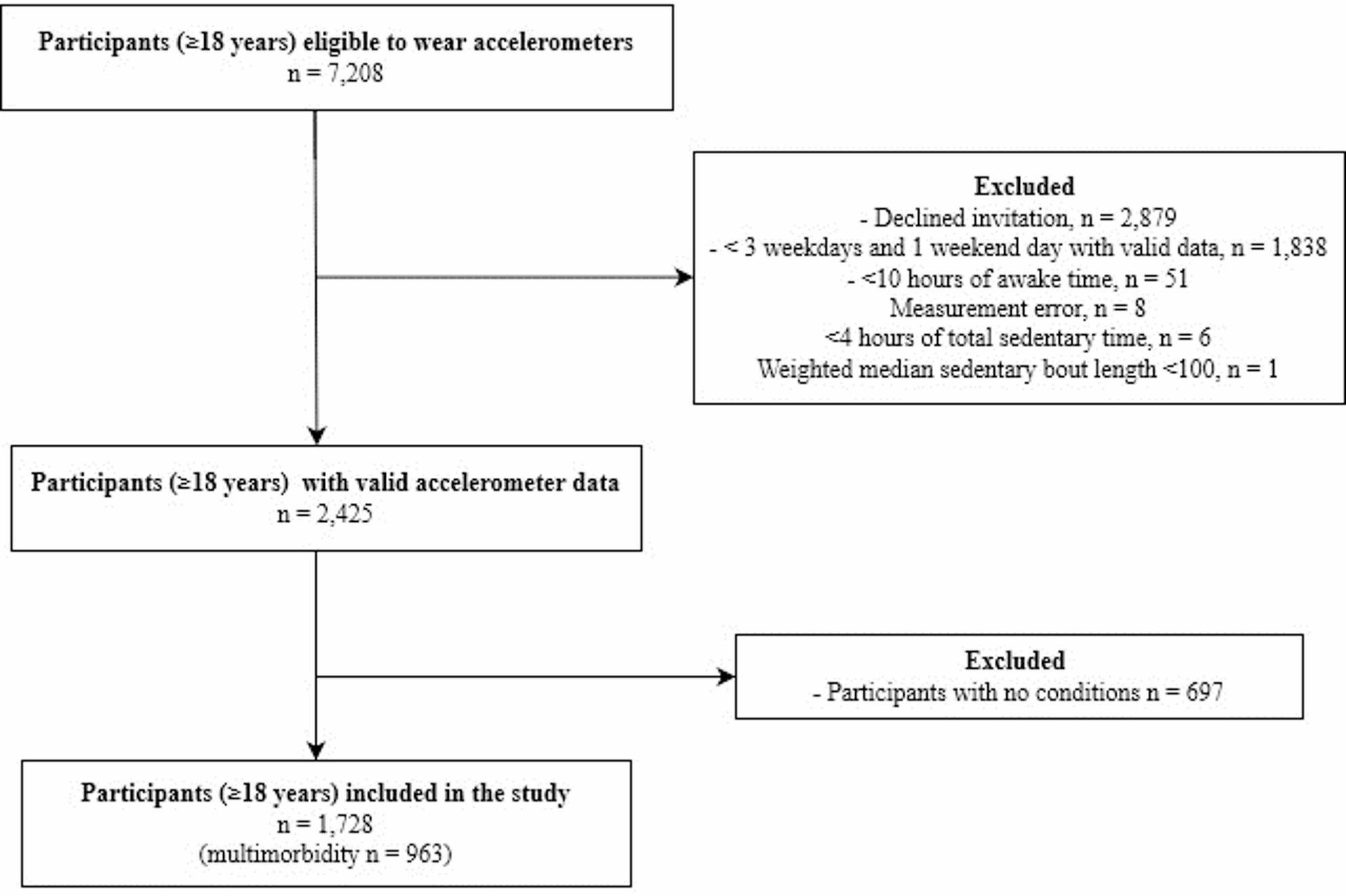
Shows the flow of particpants

The analytical sample comprised 1,728 adults with ≥ 1 medical condition, of whom 963 met the definition of multimorbidity (Fig. 1).

Participants with multimorbidity were older than those with one medical condition (mean age 63.0 vs. 58.3 years), while the sex distribution was similar (female: 56.2% vs. 54.2%). Compared with participants with one medical condition, the multimorbidity group more often had obesity (BMI ≥ 30: 33.8% vs. 23.7%), more frequently reported reduced mental well-being (20.7% vs. 10.7%), and a larger proportion were retired (55.7% vs. 37.7%) (Table 1).

**Table 1:** Clinical characteristics, sociodemographic factors, and accelerometer-assessed sedentary behaviour among participants with ≥1 medical condition (n=1,728), overall and stratified by 1 medical condition versus multimorbidity^1^

	1 medical condition	Multimorbidity^1^	Total
N	765	963	1728
Age, mean (SD)	58.3 (15.0)	63.0 (12.9)	61.0 (14.1)
Sex			
Female	415 (54.2%)	541 (56.2%)	956 (55.3%)
BMI			
Underweight/normal weight	282 (37.4%)	259 (27.6%)	541 (32.0%)
Overweight	295 (39.1%)	362 (38.6%)	657 (38.8%)
Obese class I	128 (17.0%)	216 (23.1%)	344 (20.3%)
Obese class II	42 (5.6%)	77 (8.2%)	119 (7.0%)
Obese class III	8 (1.1%)	23 (2.5%)	31 (1.8%)
Number of medical conditions			
1 condition	765 (100.0%)	-	
2 conditions	-	546 (56.7%)	-
3 conditions	-	275 (28.6%)	-
≥4 conditions	-	142 (14.7%)	-
SES (educational level)			
Primary/lower secondary	180 (23.7%)	346 (36.0%)	526 (30.5%)
Upper secondary/vocational)	247 (32.5%)	260 (27.1%)	507 (29.4%)
Higher	279 (36.7%)	291 (30.3%)	570 (33.1%)
Other	55 (7.2%)	64 (6.7%)	119 (6.9%)
Civil status			
Living with partner	619 (81.3%)	742 (77.7%)	1361 (79.3%)
Living alone	142 (18.7%)	213 (22.3%)	355 (20.7%)
Employment status			
In paid employment	416 (54.5%)	359 (37.3%)	775 (44.9%)
Unemployed/other	59 (7.7%)	68 (7.1%)	127 (7.4%)
Retired	288 (37.7%)	536 (55.7%)	824 (47.7%)
Chronic pain(lasting >6 months)			
Yes	201 (26.4%)	457 (47.8%)	658 (38.3%)
Mental well-being (WHO-5)			
Moderate to high	662 (89.3%)	731 (79.3%)	1393 (83.8%)
Reduced	79 (10.7%)	191 (20.7%)	270 (16.2%)
Total sedentary time*; mean hours/day (SD)	9.2 (1.5)	9.7 (1.7)	9.5 (1.6)
Weighted median sedentary bout length**; mean sec (SD)	236.4 (42.2)	247.5 (45.6)	242.6 (44.4)
Total active time***; median min/day (IQR)	218.01 (176.10 – 259.31)	191.45 (147.37 – 239.69)	204.48 (157.96 – 249.22)
Number of sedentary bouts****;median bouts/day (IQR)	54.71 (45.35 – 68.08)	52.14 (42.86 – 63.83)	53.14 (44.00 – 66.55)
Fragmentation index*****; mean (SD)	0.002 (0.001)	0.002 (0.001)	0.002 (0.001)

*Abbreviations*  *BMI* body mass index, *SES* socioeconomic status, *WHO-5* World Health Organization–Five Well-Being Index, *sec* seconds, *min* minutes, *IQR* interquartile range

^1^≥2 medical conditions affecting ≥2 body systems

*Total sedentary time: hours/day spent sitting, reclined, or lying. ** Weighted median sedentary bout length: duration (sec) of the bout containing the 50th percentile of total sedentary time when sedentary bouts are ordered from shortest to longest. *** Total active time: minutes/day spent in PA at light, moderate, or vigorous intensity. **** Sedentary bout: ≥10 seconds classified as sitting, reclined, or lying. ***** Fragmentation index: number of sedentary bouts divided by total sedentary time (min). All accelerometer-derived metrics were calculated for the daytime window (07:00–22:00)

### Associations with total sedentary time and weighted median sedentary bout length

Higher BMI, three or more medical conditions, living alone, and being unemployed/other or retired were associated with both greater total sedentary time and longer weighted median sedentary bout length among adults living with medical conditions. Female sex was associated with lower total sedentary time and shorter weighted median sedentary bout length. Reduced mental well-being was associated with greater total sedentary time but not with weighted median sedentary bout length (Table 2). For example, compared with underweight/normal weight, obesity class II was associated with 1.52 additional hours/day of sedentary time (95% CI 1.23–1.81) and 35.54 seconds longer weighted median sedentary bout length (95% CI 27.46–43.61), while female sex was associated with 0.23 fewer hours/day of sedentary time (95% CI − 0.37 to − 0.08) and 8.00 s shorter weighted median sedentary bout length (95% CI − 12.07 to − 3.93) (Table 2).

**Table 2:** Associations between clinical characteristics, sociodemographic factors, and sedentary behaviour outcomes among adults with ≥ 1 medical condition (*n* = 1,728). Estimates are from multivariable linear regression models (one per outcome), with age and sex forced into all models.

Dependent variable	Predictor variable	Beta value	95% CI	*p*-value
Total sedentary time (hours/day)				
	*Clinical characteristics*			
	BMI			
	Underweight/normal weight	(reference)		
	Overweight	0.33	(0.16, 0.50)	**< 0.001**
	Obese class I	0.88	(0.66, 1.10)	**< 0.001**
	Obese class II	1.52	(1.23, 1.81)	**< 0.001**
	Obese class III	1.43	(0.91, 1.95)	**< 0.001**
	Number of medical conditions			
	1	(reference)		
	2*	-	-	-
	3	0.30	(0.09, 0.51)	**0.003**
	≥4	0.28	(0.01, 0.54)	**0.049**
	Mental well-being (WHO 5 index)			
	Moderate to high	(reference)		
	Reduced	0.23	(0.01, 0.45)	0.023
	Chronic pain**	-	-	-
	*Sociodemographic factors*			
	Age***			
	18–44	(reference)		
	45–64	0.10	(-0.12, 0.32)	0.375
	65–79	0.31	(-0.01, 0.63)	0.058
	+ 80	0.46	(-0.10, 1.02)	0.066
	Sex***			
	Male	(reference)		
	Female	-0.23	(-0.37, -0.08)	**0.002**
	SES (educational level)**	-	-	**-**
	Civil status			
	Married/living with a partner	(reference)		
	Living alone	0.30	(0.09, 0.50)	**0.001**
	Employment status			
	In paid employment	(reference)		
	Unemployed/other	0.64	(0.33, 0.94)	**< 0.001**
	Retired	0.39	(0.12, 0.66)	**0.002**
Weighted median sedentary bout length (seconds)				
	*Clinical characteristics*			
	BMI			
	Underweight/normal weight	(reference)		
	Overweight	7.09	(2.18, 12.00)	**0.005**
	Obese class I	20.58	(14.54, 26.63)	**< 0.001**
	Obese class II	35.54	(27.46, 43.61)	**< 0.001**
	Obese class III	37.01	(23.28, 50.74)	**< 0.001**
	Number of medical conditions			
	1	(reference)		
	2*	-	-	-
	3	9.81	(3.90, 15.73)	**0.001**
	≥4	9.96	(2.52, 17.40)	**0.013**
	Mental well-being (WHO 5 index)**	-	-	**-**
	Chronic pain**	-	-	**-**
	*Sociodemographic factors*			
	Age***			
	18–44	(reference)		
	45–64	0.42	(-6.01, 6.86)	0.893
	65–79	4.05	(-5.06, 13.17)	0.357
	+ 80	5.93	(-9.26, 21.12)	0.402
	Sex***			
	Male	(reference)		
	Female	-8.00	(-12.07, -3.93)	**< 0.001**
	Civil status			
	Married/living with a partner	(reference)		
	Living alone	8.50	(2.79, 14.20)	**0.001**
	SES (educational level)**			
	Employment status			
	In paid employment	(reference)		
	Unemployed/other	17.57	(9.13, 26.00)	**< 0.001**
	Retired	8.56	(1.02, 16.01)	**0.017**

*Abbreviations* *BMI* body mass index, *SES* socioeconomic status, *CI* confidence interval, *WHO-5* World Health Organization–Five Well-Being Index, *variable category omitted due to *p* ≥ 0.05 in backward elimination. **variable omitted due to p-value *p* ≥ 0.05. ***forced into regression due to position as key variable

Values in bold indicate statistically significant results (*p* < 0.05)

## Discussion

Our main findings suggest that higher BMI, a greater number of medical conditions, living alone, and being unemployed/other or retired are associated with greater sedentariness among adults living with medical conditions (including a substantial proportion with multimorbidity). Female sex was associated with lower sedentariness. Reduced mental well-being was associated with higher total sedentary time, but not with weighted median sedentary bout length.

This study extends existing device-based evidence by examining both total sedentary time and sedentary accumulation using dual accelerometry in a large population of adults living with medical conditions, including many with multimorbidity.

Our findings align with previous research linking higher BMI, living alone, and unemployment to increased SB in the general adult population [17, 40, 41]. However, direct comparisons across studies remain challenging because device-based and self-reported estimates of SB may differ and because SB definitions and processing vary [19]. In our study, the association with BMI was consistent across both sedentary volume and accumulation (Table 2), suggesting that higher BMI may be associated with both higher sedentary time and more prolonged sedentary accumulation.

While some studies have focused on the association between multimorbidity and SB [14, 15, 22, 42], there is a lack of studies that have given specific attention to the association between the number of conditions and SB. We found that higher numbers of medical conditions (three and ≥four) were associated with increased sedentary time and longer weighted median sedentary bout length, whereas two medical conditions were not. Kandola et al. (2020) reported results similar to ours in a large population-based sample of older adults (*n* = 6,903) 11. In their study, after adjustment for sex, age, educational level, financial strain, and employment status, the authors found significantly higher SB with three and four conditions compared with the reference group (zero conditions) [11]. Notably, as in our study, their results did not support an association for two conditions. Taken together, these findings may suggest a non-linear association between number of medical conditions and SB. However, neither Kandola et al. nor we had access to data on disease severity or functional limitations, which may be related to SB [43, 44]. Consequently, it is possible that participants with two medical conditions had less severe disease and/or lower functional impact than those with three or more conditions, providing a plausible explanation for this pattern.

Evidence supports that women are less physically active than men when the comparison is based on meeting physical activity guidelines [45]. However, we found that women were less sedentary than men, which contrasts with findings from some accelerometer-based studies in adults without chronic conditions [46, 47] and highlights that patterns observed in healthier populations may not translate directly to adults living with medical conditions [48]. Our findings may be explained by the fact that 40% of those invited to wear accelerometers declined to participate (Fig. 1), and participants who agreed to participate may differ systematically from non-participants, potentially influencing observed sex differences in SB.

We found no association between higher age and greater SB. This finding is partially supported by evidence from studies conducted in general populations, as reported in systematic reviews [17, 18, 30]. The absence of an overall association does not exclude effect modification or non-linear age patterns, which should be examined in future studies. For instance, Rhodes et al. (2012) identified an association between higher age and greater SB in some contexts, particularly TV viewing, but not for others like reading [30]. However, these studies predominantly assessed SB using self-reported measures, differed in SB definitions (or did not define it at all), or measured SB using only one accelerometer [49–52]. In our study, we used the widely adopted SB definition from the Sedentary Behavior Research Network (SBRN) [1]. Such context-specific patterns, together with variation in SB definitions and measurement approaches across studies, may contribute to discrepant findings.

Unlike us, previous studies have reported an association between pain and SB [31]. In our study, chronic pain was assessed using a single dichotomous item (pain ≥ 6 months), which does not capture severity or interference; therefore, we could not determine whether pain acted as a functional limitation that might explain higher SB in some individuals.

### Strengths and limitations

A key strength of this study is the use of accelerometry to assess SB, which provides more accurate estimates than self-report [53]. In addition, the dual-accelerometer protocol reduces misclassification of non-ambulatory activities as SB, strengthening internal validity [54].

The cross-sectional design precludes causal inference and does not allow assessment of whether the observed associations with SB reflect reductions in LPA versus MVPA, or the testing of potential causal pathways (e.g., mediation by LPA or MVPA). Future longitudinal and intervention studies should examine these pathways.

The included correlates were selected a priori based on clinical and public health relevance and availability in LOFUS. Consequently, environmental and neighbourhood-level determinants (e.g., built-environment attributes), which may influence SB beyond individual-level factors, could not be examined in the present analysis and should be incorporated in future studies [55].

Participation in the accelerometer assessment was incomplete, and exclusions due to insufficient valid data were substantial (40% declined participation; 26% excluded), which may have introduced selection bias if those included differed systematically from non-participants in clinical characteristics, sociodemographic factors, or SB. Furthermore, LOFUS is a regional, household-based cohort and accelerometer participation was voluntary, which may limit generalisability to other populations.

Age is closely related to the accumulation of medical conditions and may modify associations with SB; future studies should therefore consider age-stratified analyses or interaction modelling. Several exposures, including questionnaire-based (self-reported) medical conditions and chronic pain, may be subject to misclassification compared with registry- or medical record–based ascertainment, which could attenuate associations.

Finally, the clinical relevance of differences in SB outcomes remains uncertain, as Minimal Clinically Important Difference (MCID) values for SB are not well established in adults living with medical conditions, including multimorbidity [38]. In this study, multimorbidity was operationalised based on available LOFUS data and previously used classification approaches [28, 29]; newer consensus recommendations were published after LOFUS data collection [56], and some misclassification of multimorbidity burden is possible. We also lacked measures of mobility limitations, which may help explain associations between SB and multimorbidity [57].

### Clinical implications

Adults living with medical conditions often have repeated contact with healthcare services, providing practical opportunities to address SB within routine care. Although the cross-sectional design does not support causal conclusions or prescriptive targeting, the identified correlates may help clinicians remain attentive to sedentariness and prompt assessment and counselling about movement behaviours. Specifically, higher BMI, lower mental well-being, living alone, unemployment/retirement, and a higher number of medical conditions were associated with greater sedentariness, suggesting that these characteristics may indicate individuals who are more likely to be highly sedentary.

Importantly, these findings should be viewed as hypothesis-generating. Evidence from experimental studies indicates that interrupting prolonged sitting with brief activity breaks and modest increases in PA can improve cardiometabolic outcomes [8, 9]. Future longitudinal and intervention studies in adults with medical conditions, including those living with multimorbidity, should test whether such strategies are feasible and effective, and whether effects differ across clinical and sociodemographic subgroups.

## Conclusion

We examined whether clinical characteristics and sociodemographic factors were associated with accelerometer-assessed SB among adults living with medical conditions, including a substantial proportion with multimorbidity. Higher BMI, a greater number of medical conditions, living alone, and being unemployed/other or retired were associated with greater sedentariness across both total sedentary time and sedentary accumulation, while reduced mental well-being was associated with higher total sedentary time. Female sex was associated with lower sedentary time and shorter weighted median sedentary bout length. These findings add device-based evidence on SB correlates in adults living with medical conditions and may help inform hypotheses and the design of future longitudinal and intervention studies. In clinical settings, these correlates may prompt attention to SB and support conversations about movement behaviours, but causal inferences cannot be drawn from this cross-sectional study.

## Supplementary Information

Supplementary Material 1.

Supplementary Material 2.

## Data Availability

The datasets generated and analyzed during the current study are available from the corresponding author on reasonable request.

## Abbreviations

SB: Sedentary Behavior
PA: Physical Activity
SES: Socioeconomic Status
BMI: Body Mass Index
STROBE: Strengthening the reporting of observational studies in epidemiology
LOFUS: The Lolland-Falster Health Study
WHO: World Health Organization
SD: Standard Deviation
Sec: Seconds
Min: Minutes
