# Supplementary material for: The relationship between clinical characteristics, sociodemographic factors and sedentary behavior in adults with chronic conditions: a cross-sectional study from the Lolland-Falster Health Study
Source: Eur Rev Aging Phys Act. 2026 Apr 13;23:17. doi: 10.1186/s11556-026-00407-z (PMC13217874; doi:10.1186/s11556-026-00407-z)
Supplement: Supplementary file 2 — Supplementary Material 2. [file 11556_2026_407_MOESM2_ESM.docx]

**Supplementary file 2. Classification of medical conditions**

This supplementary file describes the procedure used to classify self-reported medical conditions in the Lolland-Falster Health Study (LOFUS) and assign them to body system groups.

In the LOFUS questionnaire, participants selected medical conditions from 18 response options. Option 18 (“Other”) was a free-text field where participants could report additional conditions not covered by the predefined options.

**Processing of free-text “Other” responses**

Free-text entries (“Other”) reported by LOFUS participants were reviewed and classified using a predefined procedure developed in connection with a previous LOFUS study (Tang et al. 2020 ). Specifically:

- Free-text entries recorded from 8 February 2016 to 7 March 2019 were reviewed by Author A and a research assistant with a medical/health-science background, using the criteria below.
- Free-text entries recorded after 7 March 2019 were reviewed by Author B and Author C using the same procedure. Any discrepancies were resolved through discussion until consensus was reached.

Inclusion criteria (eligible for classification)

- Clearly specified diagnoses (e.g., atrial fibrillation).
- Conditions that clearly indicated a medical condition and its anatomical/clinical domain (e.g., gastrointestinal disease).

Exclusion criteria (not classified as medical conditions)

- Past/resolved conditions (e.g., conditions explicitly stated as former).
- Symptoms or complaints not defined as diseases (e.g., dizziness).
- Risk factors rather than diagnoses.

**Body system grouping**

Eligible conditions were assigned to one of ten body system groups based on similarities in treatment, clinical manifestation, and/or organisation in the healthcare system: lung, musculoskeletal, endocrine, mental, cancer, neurological, gastrointestinal, cardiovascular, kidney, and sensory organs (Table below)

| **Chronic condition group** | **Lung** | **Musculoskeletal** | **Endocrine** | **Mental** | **Cancer** |
| --- | --- | --- | --- | --- | --- |
|  | ***Standardized response option used in the LOFUS survey*** | | | | |
|  | - Asthma | - Osteoarthritis | - Diabetes | - Anxiety | - All cancer types |
|  | - Chronic bronchitis, emphysema, chronic obstructive pulmonary disease (COPD) | - Rheumatoid arthritis |  | - Depression |  |
|  |  | - Spinal hernia or other spinal diseases |  |  |  |
|  | ***Conditions reported and coded from the “Others” disease category*** | | | | |
|  |  |  |  |  |  |
|  |  |  |  |  |  |
|  |  |  |  |  |  |
|  |  |  |  |  |  |
|  |  |  |  |  |  |
| **Chronic condition group** | **Neurological** | **Gastrointestinal** | **Cardiovascular** | **Kidney** | **Sensory organs** |
|  | ***Standardized response option used in the LOFUS survey*** | | | | |
|  | - Migraine or frequent headaches |  | - Acute myocardial infarction | - Kidney disease | - Allergy (not asthma) |
|  |  |  | - Atherosclerosis in the heart |  |  |
|  |  |  | - Angina pectoris |  |  |
|  |  |  | - Blood clot (thrombosis) in the leg - Hypertension* |  |  |
|  | ***Conditions reported and coded from the “Others” disease category*** | | | | |
|  | ***Conditions reported and coded from the “Others” disease category that did not fit under any of the ten chronic condition groups (excluded).*** | | | | |
|  |  |  |  |  |  |
|  |  |  |  |  |  |
|  |  |  |  |  |  |

*****hypertension was included in the present study but excluded from the study by Tang et. al 2020 <https://pubmed.ncbi.nlm.nih.gov/33415082/>
